# Genetic variation is associated with PTSD risk and aversive memory: Evidence from two trauma-Exposed African samples and one healthy European sample

**DOI:** 10.1038/s41398-018-0297-1

**Published:** 2018-11-22

**Authors:** Sarah Wilker, Anna Schneider, Daniela Conrad, Anett Pfeiffer, Christina Boeck, Birke Lingenfelder, Virginie Freytag, Vanja Vukojevic, Christian Vogler, Annette Milnik, Andreas Papassotiropoulos, Dominique J.-F. de Quervain, Thomas Elbert, Stephan Kolassa, Iris-Tatjana Kolassa

**Affiliations:** 1 Clinical & Biological Psychology, Ulm University, Ulm, Germany; 2 Clinical Psychology and Neuropsychology, University of Konstanz, Konstanz, Germany; 3 Division of Molecular Neuroscience, University of Basel, Basel, Switzerland; 4 Transfaculty Research Platform Molecular and Cognitive Neurosciences, University of Basel, Basel, Switzerland; 5 Department Biozentrum, Life Sciences Training Facility, University of Basel, Basel, Switzerland; 6 Psychiatric University Clinics, University of Basel, Basel, Switzerland; 7 Division of Cognitive Neuroscience, University of Basel, Basel, Switzerland; 8 SAP Switzerland AG, Tägerwilen, Switzerland

## Abstract

The probability to develop posttraumatic stress disorder (PTSD), characterized by vivid, intrusive emotional memories of the encountered traumatic events, depends - among other factors - on the number of previous traumatic experiences (traumatic load) and individual genetic vulnerability. So far, our knowledge regarding the biological underpinnings of PTSD is relatively sparse. Genome-wide association studies (GWAS) followed by independent replication might help to discover novel, so far unknown biological mechanisms associated with the development of traumatic memories. Here, a GWAS was conducted in *N* = 924 Northern Ugandan rebel war survivors and identified seven suggestively significant single nucleotide polymorphisms (SNPs; *p* ≤ 1 × 10^−5^) for lifetime PTSD risk. Of these seven SNPs, the association of rs3852144 on chromosome 5 was replicated in an independent sample of Rwandan genocide survivors (*N* = 370, *p* < .01). While PTSD risk increased with accumulating traumatic experiences, the vulnerability was reduced in carriers of the minor G-allele in an additive manner. Correspondingly, memory for aversive pictures decreased with higher number of the minor G-allele in a sample of *N* = 2698 healthy Swiss individuals. Finally, investigations on *N* = 90 PTSD patients treated with Narrative Exposure Therapy indicated an additive effect of genotype on PTSD symptom change from pre-treatment to four months after treatment, but not between pre-treatment and the 10-months follow-up. In conclusion, emotional memory formation seems to decline with increasing number of rs3852144 G-alleles, rendering individuals more resilient to PTSD development. However, the impact on therapy outcome remains preliminary and further research is needed to determine how this intronic marker may affect memory processes in detail.

## Introduction

Traumatic experiences such as war, terror, or natural disasters can lead to the development of posttraumatic stress disorder (PTSD), a disorder characterized by extremely vivid emotional memories of the traumatic events experienced. PTSD symptomatology is associated with severe individual suffering, impairments in daily functioning^1,2^, increased physical health risk^3–5^, and suicidality^6^. The risk of PTSD development increases with the number of different traumatic event types experienced (traumatic load)^7–10^, and approaches 100% at extreme levels of traumatic load^11^. Nevertheless, substantial inter-individual differences can be observed at lower levels of traumatic load, which underscores the role of individual risk factors in the aetiology of PTSD. Similarly, individuals vary in their response to exposure-based psychotherapies for PTSD. Although these treatments are considered very effective for PTSD^12,13^, approximately one third of trauma survivors still present with clinically significant symptoms after therapy^14–16^. A deeper knowledge about individual predispositions related to PTSD development or lower treatment responsiveness might help to identify individuals at risk early, allocate therapeutic resources accordingly, and finally yet importantly, to personalize treatments according to the individual needs of trauma survivors.

The heritability of PTSD development following trauma exposure has been estimated to be at least 30–40%^17–20^, indicating a substantial influence of genetic risk factors in the development of PTSD. Until now, over 100 candidate-gene association studies^2,21,22^ and nine GWAS have been published^23–31^ (for a recent review see^32^). While candidate-gene approaches are driven by a priori hypotheses on PTSD risk, genome-wide association studies (GWAS) represent an untargeted approach, able to detect new molecular mechanisms underlying PTSD development^22^. Yet, the simultaneous testing of millions of single nucleotide polymorphisms (SNPs) requires correction for multiple comparisons to avoid Type I errors, and the replication of significant findings in independent study cohorts is needed to assure results’ reliability. The largest PTSD GWAS to date was conducted by the Psychiatric Genomics Consortium for PTSD and showed a substantial overlap of PTSD risk loci with schizophrenia, yet none of the tested variants reached genome-wide significance^25^. Challenges of such large-scale analyses include disparities of the investigated populations regarding their ancestry^25^ and the measures applied to assess PTSD^33^. Further, inconsistencies in the assessment of trauma exposure make it difficult to include the environmental factor traumatic load in large-scale GWAS. However, the identification of genetic markers associated with the risk to develop PTSD requires an adequate assessment and due consideration of traumatic load, which accounts for a large proportion of the variability in PTSD vulnerability^25^. Therefore, studies with smaller samples exposed to the same conflict, a standardized and systematic assessment of PTSD symptomatology and traumatic load as well as its consequent inclusion into analyses might also advance our understanding of PTSD and complement the results from large-scale GWAS, even though presenting with lower statistical power.

Since PTSD has been conceptualized as a disorder of pathological memory formation of the traumatic experiences^34–40^, the investigation of genetic factors related to memory performance in healthy individuals can give important insights in the genetic underpinnings of PTSD (for a review see^2^). In previous studies, we were able to identify genetic factors associated both with healthy memory performance and PTSD risk^41–44^, indicating a possible overlap in the genetic underpinnings of regular emotional memories and the development of extremely distressing trauma memories. In this line, another key question in PTSD genetic research is whether previously identified PTSD risk genes also influence the modification of traumatic memories by trauma-focused exposure-based psychotherapy. This would underscore the clinical relevance of genetic risk factors for PTSD, which normally have small effect sizes. First efforts have been made to examine the influence of reported risk genes (e.g., *FKBP5*, *SLC6A4*, and *BDNF*) on therapeutic treatment outcomes. Despite small sample sizes, significant associations between these risk variants and the modification of trauma memories by means of exposure-based psychotherapy were found^45–47^.

In this study we investigated genetic markers underlying PTSD risk in two independent African samples, including the influence of traumatic load in all analyses stages. We furthermore tested for significant associations of the discovered genetic markers with memory performance in healthy Europeans and with psychotherapy success in a subsample of Northern Ugandan rebel war survivors who were diagnosed with PTSD and treated with Narrative Exposure Therapy (NET^48^).

## Materials and methods

### Samples

#### Ugandan discovery sample

Participants were survivors of the rebel war in Northern Uganda, who were severely affected by the atrocities committed by the rebel group Lord’s Resistance Army (LRA) (e.g., abductions, forced recruitment, killings, mutilations, and sexual violence) who was active for about 20 years since 1987. Data collection took place in the former internally displaced people (IDP) camps of Anaka, Pabbo (Amuru District) and Koch Goma (Nwoya District), and in re-settled communities and villages of Gulu District, Northern Uganda (details on the recruitment are described in the Supplement). Inclusion criteria were an age of 18 years and above, absence of signs for alcohol or substance addiction, absence of severe acute psychotic symptoms and no current intake of psychotropic medication. To control for a potential inflation of genetic effects by related participants, we furthermore asked only one family member per household to participate. A total number of *N* = 1148 participants of this ongoing study were interviewed and genotyped at the 2017-01-05 data freeze. Out of these *N* = 17 were excluded due to missing behavioural data. Furthermore, *N* = 207 participants were excluded based on the following criteria (see Supplement for more details): inconsistency between reported and genetically inferred sex, genome-wide call rate < 95%, unusual ancestry genetic background (Bayesian Clustering outlier detection^49^), cryptic relatedness (IBD sharing pi-hat > 0.2; one-sample of each detected pair was excluded) and Bayesian Clustering outlier detection on genome-wide call and heterozygosity rates^49^, leading to a sample of *N* = 924 (see Table 1 for a demographic overview).

**Table 1:** Demographic and clinical data overview of the Ugandan discovery sample, Rwandan replication sample and Ugandan therapy sample

	Ugandan discovery sample (*N* = 924)	Rwandan replication sample (*N* = 371)	Ugandan therapy sample (*N* = 90)
*N* female (%)	501 (54.22)	179 (48.24)	55 (61.11)
Mean age (s.d.)	31.26 (10.74)	34.65 (5.88)	31.33 (9.22)
Mean traumatic load (s.d.)	26.35 (8.95)	11.88 (5.20)	37.00 (6.57)
*N* Lifetime PTSD diagnosis (%)	644 (69.70)	263 (70.89)	90 (100)
*N* Current PTSD diagnosis (%)	195 (21.10)	158 (42.59)	90 (100)
Mean PDS Score (t1) (s.d.)	4.67 (6.28)	13.50 (10.72)	16.82 (4.77)

In the Ugandan discovery sample and the Ugandan therapy sample a 62-item event list was used to assess traumatic load, while in the Rwandan replication sample a 36-item event list was applied, hence, lower levels of trauma load in the Rwandan sample result from the applied event-list

*PTSD* = posttraumatic stress disorder, *PDS* = Posttraumatic Stress Diagnostic Scale

Importantly, the majority of the individuals (*N* = 180) were excluded due to indications of first or second-degree relationships with other participants in the sample (pi-hat > 0.2). This might be due to the fact that a large number of participants were sampled in former IDP camps and re-settled communities, where family members are frequently living together, but in separate households. As the resulting sample still comprised a large number of participants, who are likely third-degree relatives to other individuals in the sample (pi-hat > 0.1), we further replicated our analyses applying this more stringent threshold, resulting in a sample of *N* = 799 individuals. Further we applied the following SNP inclusion criteria: minor allele frequency > .05, SNP call rate > .95, non-deviance from Hardy-Weinberg equilibrium > 0.05 and only considered autosomal SNPs, resulting in 654,099 SNPs used in the downstream analysis.

#### Rwandan replication sample

The sample included *N* = 409 survivors of the Rwandan genocide who all lived in the refugee settlement Navikale, Uganda. Study inclusion criteria and applied quality controls were the same as for the Ugandan discovery sample (see Supplement for details on recruitment and quality criteria applied). Further cases with missing data regarding lifetime PTSD diagnoses and traumatic load were dropped, leading to a sample of *N* = 371 (see Table 1). Contrary to the Ugandan sample, the Rwandan sample only contained a small proportion of relatives. Therefore, the threshold of pi-hat > 0.2 was sufficient.

#### Healthy Swiss sample

The subjects of this cohort represent subsets of two ongoing studies, which were described previously^44,50^. The purpose of these ongoing studies is to identify biological correlates of cognitive performance by using genetics, electroencephalography (EEG study) and imaging techniques (fMRI study) in healthy young adults from the general population. The reported data is based on the data freeze 2015-09-23. Participants indicating any lifetime neurological or psychiatric disease or current medication intake (except oral contraception) were excluded from study participation. Further, we excluded subjects with missing phenotypic data and genetic outliers (see Supplement). After exclusion, the combined sample comprised *N* = 2699 healthy individuals (1741 women, *M*_age_ = 22.51, SD_age_ = 3.47). Of those, 1580 individuals took part in the EEG study (1062 women, *M*_age_ = 22.55, SD_age_ = 3.59) and 1119 participated in the fMRI study (679 women, *M*_age_ = 22.46, SD_age_ = 3.29), and will in the following be referred to as *EEG sample* and *fMRI sample*.

#### Ugandan therapy sample

This sample of *N* = 90 individuals (see Table 1) represents a subset of participants of the discovery sample, who fulfilled the diagnostic DSM-IV-TR^51^ criteria for a current PTSD at the time of the first interview and were offered a treatment with NET^48^. NET is an exposure-based short-term therapy for survivors of multiple traumatic experiences who suffer from PTSD. The treatment aims at transforming the defragmented memories of the traumatic experiences into a coherent and chronological narrative. The therapy was performed by trained local counselors under close supervision of expert psychologists in form of weekly supervision meetings, case discussions and detailed case documentations. In the first session, the lifeline exercise is performed in order to gain a chronological overview of the client’s life story. In the following sessions, the therapist instructs the client to chronologically report on significant life events, with a particular focus on imaginal exposure of the most traumatic experiences. Hereby the client is encouraged to share his or her emotional, behavioural, cognitive, and physiological reactions experienced during the traumatic event. The clients should relive these emotional reactions in a controlled and secure environment in order to contextualize the fear memories^48^. Participants received on average 12 sessions of NET that lasted 90–120 min and generally took place twice a week. Diagnostic assessments were carried out before treatment (t_1_), and four (t_2_) and 10 months (t_3_) after the end of treatment.

Table 1 provides an overview of the demographic characteristics and clinical data of the three trauma-exposed samples investigated.

### Materials and study procedure

Study procedures were approved by the Institutional Review Board of Gulu University, Uganda, the Ugandan National Council for Science and Technology and the ethics committee of the German Psychological Society (Deutsche Gesellschaft für Psychologie, DGPs) for the Ugandan samples, the University of Konstanz, Germany, and the University of Mbarara, Uganda, for the Rwandan sample, and by the ethics committees of the Cantons Basel-Stadt and Basel-Landschaft for the Swiss samples. All participants provided written informed consent prior to study participation.

Demographic and clinical data in the African samples were assessed via structured clinical interviews. The Posttraumatic Stress Diagnostic Scale (PDS^52^) was used to determine each participant’s PTSD status according to DSM-IV-TR^51^ covering lifetime PTSD diagnosis, current PTSD diagnosis (used to assign participants to the therapy sample) and current PTSD symptom severity. The reliability and validity of the translated PDS has been assured in previous studies with Ugandan^53^ and Rwandan trauma survivors^54^. Cronbach’s alpha in this study was .92 for the Ugandan and .92 for the Rwandan study cohort. Diagnostic interviews were conducted by expert psychologists with the help of trained interpreters and trained local counselors under supervision of psychologists with specialization in psychotraumatology. All diagnostic instruments were translated into the local languages (Kinyarwanda, Rwandan replication sample, and Luo, Ugandan discovery and therapy sample) according to scientific standards, following a procedure of blind translation, back-translation and group discussions by independent interpreters.

An event-list comprising 62 items (Ugandan discovery and therapy sample), or 36 items (Rwandan replication sample), was used to assess traumatic load, which was included as covariate in all analyses in the African samples. The event-list of the Rwandan replication sample comprised general traumatic experiences (e.g., rape, natural disaster), and events specific for the context of armed conflicts (e.g., fighting in combat, bomb attack). This event-list was extended to include further atrocities specific to the war of the LRA (e.g., forced to eat human flesh, forced to cut off lips and ears), resulting in an event-list with 62 items for the Ugandan discovery and therapy sample, respectively. For each event, participants were asked whether it ever happened to them. Supplementary Table 1 provides an overview of the ten most frequently experienced events in each of the three groups. Previous studies indicated that the sum score of different traumatic event types experienced represents a reliable, valid and economic assessment of traumatic load^55,56^.

Participants of the healthy Swiss sample participated in a picture task in which they were presented with 24 neutral, 24 positive and 24 negative photographs for 2.5 s each, and in quasi-randomized order (a maximum of four pictures with same valence in a row). Pictures were obtained from the International Affective Picture System (IAPS^57^); neutral pictures were additionally taken from in-house standardized picture sets in order to equate the picture sets for visual complexity and content (e.g., human presence). Subjects were instructed to rate the valence (neutral, positive, negative) and arousal (low, medium, high) immediately after seeing each picture at a three-point scale (Self-Assessment Manikin; SAM). For the unannounced free recall task, 10 min after the presentation, participants were instructed to describe in writing with a few words all pictures they could recall. A picture was counted as correctly recalled if two independent raters could identify the presented picture based on the subject’s picture description. In case of a mismatch between the two raters (i.e., only one of the two raters judged the picture as correctly recalled, but the other did not), a final decision on whether the picture was successfully recalled was made by an independent blinded third rater. A more detailed description of the picture task has been reported in previous studies^44,50^.

For the genetic analyses, saliva samples were collected with Oragene DNA self-collection kits (DNA Genotek Inc., Ontario, Canada) in all study cohorts. The samples were shipped to the Transfaculty Research Platform Molecular and Cognitive Neuroscience (MCN, Basel, Switzerland) where DNA was extracted from the collected saliva samples using standard procedures. Subjects were individually genotyped using the Affymetrix Human SNP-array 6.0 and samples were processed as described in the Genome-Wide Human SNP Nsp/Sty 6.0 User Guide (Affymetrix, Santa Clara, California, USA).

### Statistical procedures

First, a GWAS was performed in the Ugandan discovery sample using PLINK software version 1.07^58^. In total, 654,099 autosomal SNPs were included in the logistic regression analyses, testing for an additive genetic effect on lifetime PTSD risk. Genotyping was done at three different time periods (genotyping-batch). We included traumatic load, sex, age and genotyping-batch (dummy-coded) as covariates. Homogeneity in ancestral background was ensured by excluding participants from the sample (cf. Supplement); ancestry was hence not included as additional covariate. Only traumatic load and genotyping-batch showed significant effects on lifetime PTSD risk and were hence included as covariates in subsequent single SNP analyses, which were conducted to follow-up all SNPs that surpassed the suggestive significance GWAS threshold (*p* ≤ 1 × 10^–5^; e.g.,^30^). For these SNPs, we individually tested whether the inclusion of a gene × environment interaction effect would increase the model fit according to Akaike’s Information Criterion^59^. Statistical significance was determined by calculating likelihood ratio (LR) tests of nested models^60^. In order to replicate the results from the Ugandan discovery sample, the same logistic regression models were fitted in the Rwandan replication sample. In this study genotyping was done at a single time period, therefore genotyping-batch was not considered as a covariate in the models. We furthermore tested whether the SNPs replicated in the publicly available PTSD GWAS of African American samples (Psychiatric Genomics Consortium; http://www.med.unc.edu/pgc/results-and-downloads). All analyses were performed in the statistical environment R^61^ version 3.4.1, using the R package ‘GenABEL’ version 1.8.0^62^.

For SNPs reaching suggestive significance in the GWAS performed in the Ugandan discovery sample and being replicable in the Rwandan sample, we furthermore planned for genetic association tests with memory of emotionally arousing pictures in the healthy Swiss sample using linear regression models. Standardized betas are reported to express the strength of association. We regressed out potential confounding effects due to age, sex, genotyping-batch and change of the experimental environment during the free recall in the fMRI sample. In line with the analyses on lifetime PTSD risk, an additive genetic effect was assumed.

To investigate if associated PTSD risk markers also influence PTSD treatment response (i.e., changes in current PTSD symptom severity over time) linear mixed effect models were calculated in the Ugandan therapy sample using R package ‘nlme’ version 3.1.120^63^. Linear mixed models are particularly suited for longitudinal data as they can model the underlying correlational structure of repeated measurements and can easily deal with missing data. The model included the PDS sum score as the outcome variable, time as a within-subject fixed factor, genotype as a between-subject fixed factor, and the interaction genotype × time, representing a potential effect of genotype on treatment outcome. The predictor variable time was factorized to be able to test for non-linear symptom reduction. Additionally, the covariates traumatic load and genotyping-batch were included as additional fixed effects and participants were modeled as a random effect, with random intercepts for each participant. In case of a significant genotype × time interaction, planned general linear hypotheses were calculated as post-hoc tests for linear mixed effect models, using the R package ‘multcomp’ version 1.4.6^64^. We planned for three tests, investigating the influence of genotype on changes in PDS symptom score from before treatment to the 4-months follow-up (t_1_-t_2_), between the 4-months and the 10-months follow-up (t_2_-t_3_) and from pre-treatment to the 10-months follow-up (t_1_-t_3_). *P*-values were adjusted for multiple comparisons following the Holm procedure.

To assure that genotype groups did not differ in demographic and clinical data before treatment, Fisher’s exact test was applied for count data, while for continuous data a one-way analysis of variance was used. In case that residuals were non-normally distributed, a Kruskal–Wallis H test was performed. To be consistent with the previous investigations on PTSD risk, an additive genetic effect was assumed. As model residuals did not meet the assumption of normality, statistical significance was evaluated by means of permutation tests^65^ using 10, 000 random permutations. Empirical *p*-values (*p*_emp_) are reported. All tests performed in this study were two-sided with an alpha value of *p* *≤* .05 indicating statistical significance.

## Results

### Ugandan discovery sample

**Fig. 1:**
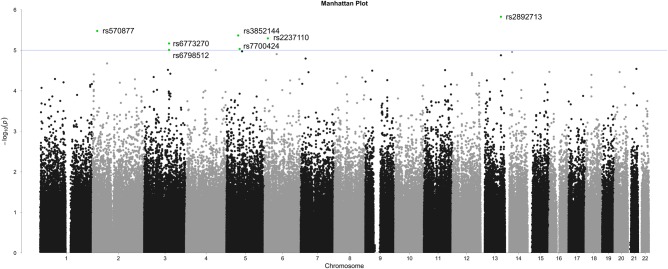
**Ugandan discovery sample**. Manhattan plot displaying genome-wide association results for the Ugandan discovery sample. Logistic regression models tested for an additive genetic effect of 654,099 autosomal SNPs and included traumatic load, sex, age, and genotyping-batch as covariates. Blue line indicates suggestive significance threshold (*p* ≤ 1 × 10^−5^)

GWAS in the Ugandan discovery sample (*N* = 924), testing for genetic associations with lifetime PTSD risk while accounting for traumatic load, sex, age and genotyping-batch as covariates, identified one SNP on chromosome 2 (rs570877), two SNPs on chromosome 3 (rs6773270 and rs6798512), two SNPs on chromosome 5 (rs3852144 and rs7700424), one SNP on chromosome 6 (rs2237110) and one SNP on chromosome 13 (rs2892713) which passed the suggestive significance threshold (p ≤ 1 × 10^–5^) (Fig. 1). All SNPs were in Hardy–Weinberg equilibrium (for more detailed SNP information see Supplementary Table 3). While the two SNPs on chromosome 3 were in complete linkage disequilibrium (*r*^2^ = 1.000), the two SNPs on chromosome 5 were unlinked (*r*^2^ = .002).

**Fig. 2:**
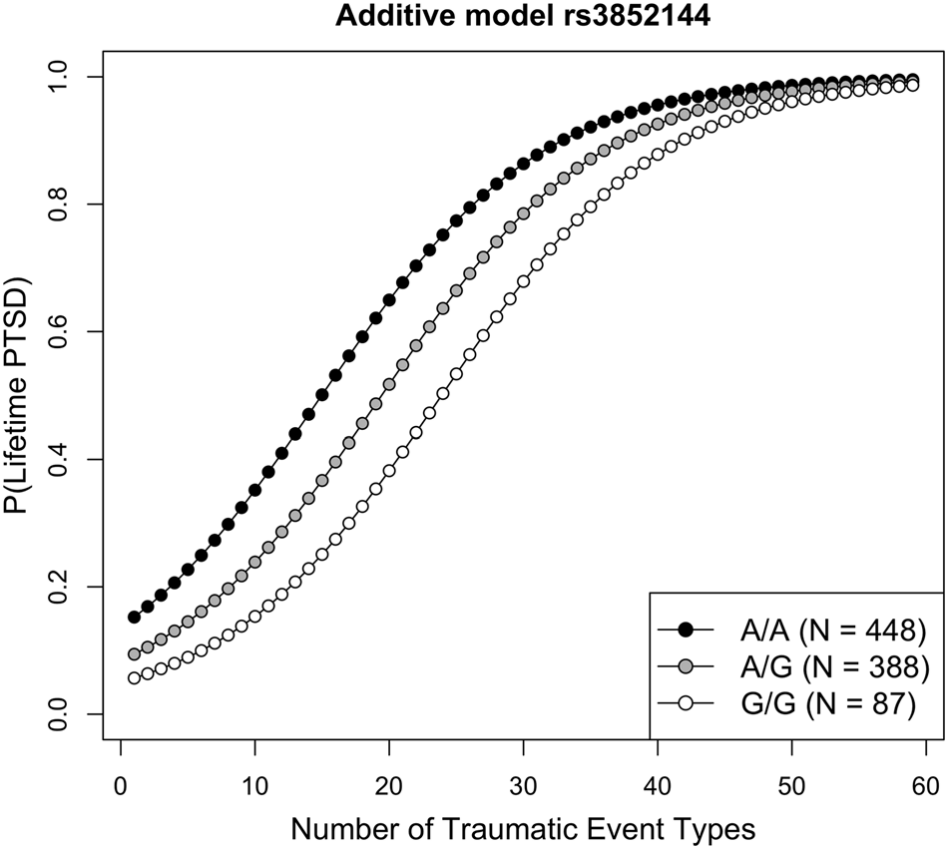
**Ugandan discovery sample**. Fitted probability values for lifetime posttraumatic stress disorder (PTSD) as a function of traumatic load are plotted separately for the three genotype groups of rs3852144 (chromosome 5). Results indicate a decreased risk for PTSD development after traumatic experiences with increasing number of the minor G-allele. Similar results were obtained in the replication sample (cf. Supplementary Figure 7)

We further found strong main effects of traumatic load (LR[1] = 169.21, *p* < .0001) and of genotyping-batch (LR[2] = 41.49, *p* < .0001) on lifetime PTSD risk, but not of age (LR[1] = 0.65, *p* = .421) or sex (LR[1] = 0.56, *p* = .456). Therefore, only traumatic load and genotyping-batch were included as covariates in subsequent single SNP analyses. For SNP rs3852144 on chromosome 5 (*N* = 923) there was a lower risk to develop PTSD with increasing number of the minor G-allele (LR[1] = 21.30, *p* = 3.931 × 10^−6^; see also Fig. 2).

For SNPs rs570877 on chromosome 2 (*N* = 924; *LR* [1] = 21.81, *p* = 3.013 × 10^–6^), rs2237110 on chromosome 6 (*N* = 915; LR[1] = 21.65, *p* = 3.268 × 10^–6^) and rs2892713 on chromosome 13 (*N* = 911; LR[1] = 23.71, *p* = 1.122 × 10^−6^) we also detected a lower risk to develop a PTSD with an increasing number of minor alleles (see Supplementary Figures 1–3).

On the contrary, for SNPs rs6773270 (N = 915; LR[1] = 21.64, *p* = 3.285 × 10^–6^) and rs6798512 on chromosome 3 (*N*=921; LR[1] = 20.80, *p* = 5.086 × 10^–6^), and for SNP rs7700424 on chromosome 5 (*N* = 916; LR[1] = 20.42, *p* = 6.224 × 10^–6^) we observed an increased PTSD risk with an increasing number of minor alleles (Supplementary Figures 4–6). Only for SNP rs570877 did the inclusion of an interaction between genotype × traumatic load improve model fit, but was not significant (*p* = .098).

To control for the high proportion of third-degree relatives in the sample and a potential inflation of genetic effects, we repeated our single SNP analyses excluding these participants by applying the more stringent threshold (pi-hat > 0.1). We replicated our initial results and observed a strong additive effect on PTSD risk for all SNPs tested (rs570877: LR[1] = 18.85, *p* *=* 1.415 × 10^−5;^ rs6773270: LR[1] = 16.16, *p* *=* 5.815 × 10^−5^; rs6798512: LR[1] = 15.25, *p* = 9.408 × 10^−5^; rs3852144: LR[1] = 19.34, *p* *=* 1.093 × 10^−5^; rs7700424: LR[1] = 21.78, *p* = 3.066 × 10^−6^; rs2237110: LR[1] = 17.59, *p* *=* 2.742 × 10^−5^; rs2892713: LR[1] = 21.68, *p* *=* 3.223 × 10^−6^).

### Rwandan replication sample

We could replicate the association of rs3852144 with lifetime PTSD risk in an independent sample of *N* = 370 subjects with complete genetic data. Again, an increase in the number of minor G-alleles was associated with a decrease in the risk to develop PTSD (LR[1] = 7.30, *p* = .007; see Supplementary Figure 7). Associations of the other identified SNPs with lifetime PTSD risk could not be replicated (rs570877: LR[1] = 0.27, *p* = .606; rs6773270: LR[1] = 1.93, *p* = .165; rs6798512: LR[1] = 2.12, *p* = .145; rs7700424: LR[1] = 1.57, *p* = .210; rs2237110: LR[1] = 0.53, *p* = .467; but the respective effects were concordant with those found in the Ugandan sample. SNP rs2892713 could not be tested for replication in the Rwandan sample as it did not meet the applied SNP quality control criteria in this cohort.

### Replication in publicly available PTSD GWAS

None of the SNPs showed significant associations with PTSD in the dataset of African Americans provided by the Psychiatric Genomics Consortium. It has to be noted that the statistical analyses for which publically available summary statistics are provided by the PGC did not include the factor traumatic load. However, for four of seven SNPs the direction of effect was similar to the one found in this study. For more details on the results, the reader is referred to Supplementary Table 4.

### Healthy Swiss sample

Investigations of rs3852144 in the healthy Swiss sample were based on *N* = 2698 individuals with complete genetic data. In the combined sample (fMRI subsample: *N* = 1118; EEG subsample EEG, *N* = 1580), we found significant associations between rs3852144 and memory for pictures with negative valence (*b* = 0.040, *t*(2696) = 2.099, *p* = .036), but not for positive (*b* = 0.03, *t*(2696) = 1.45, *p* = .146) or neutral valence (*b* = 0.02, *t*(2696) = 1.08, *p* = .282). Yet, if FDR correction was applied to account for the analyses of three dependent variables, the statistical significance threshold was not met.

**Fig. 3:**
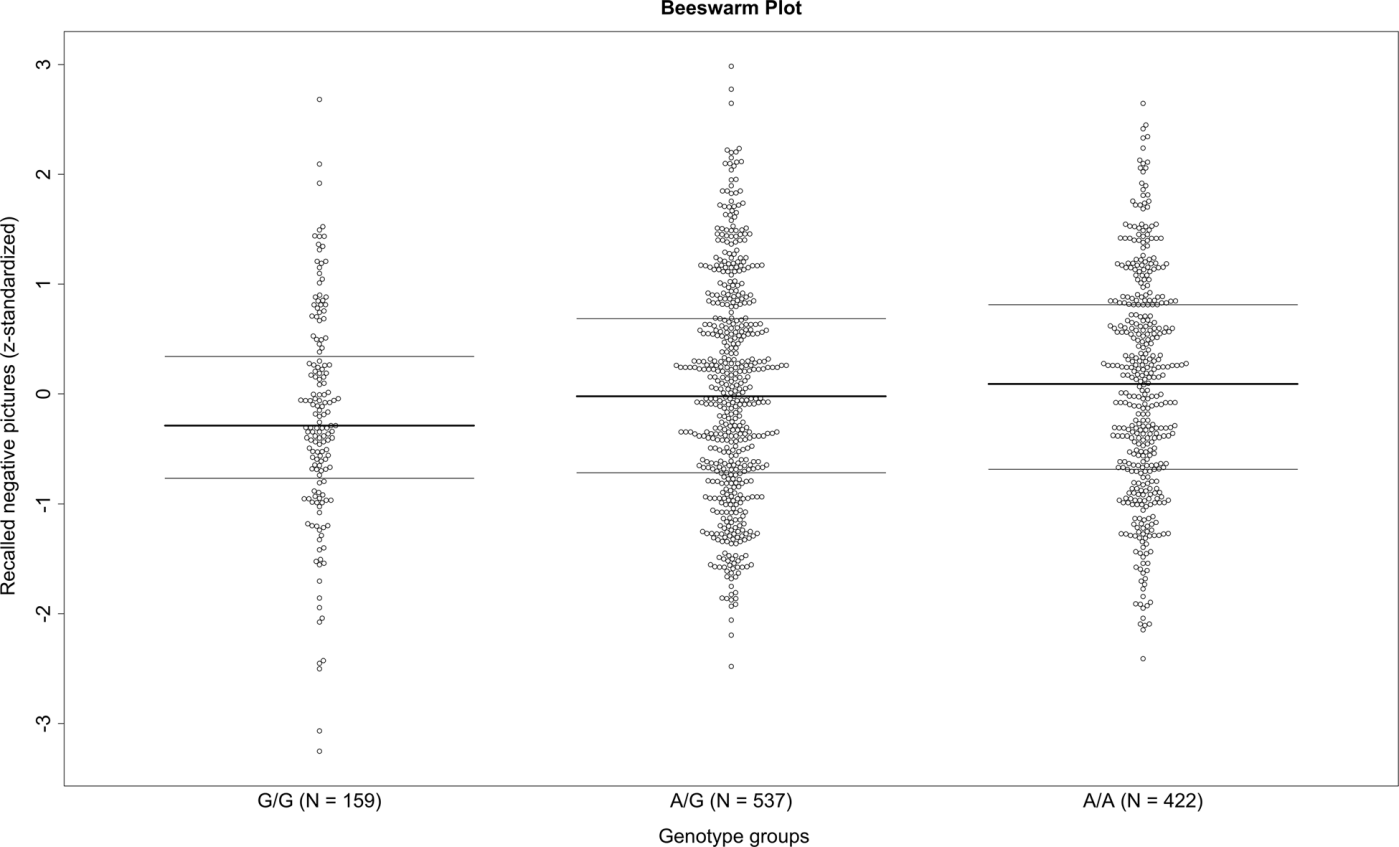
**Healthy Swiss fMRI sample.** Beeswarm plot displaying remembered negative pictures (*z*-standardized) separately for the three genotype groups of rs3852144 (chromosome 5). Consistent with the analyses on lifetime PTSD risk, results showed a decreased memory performance with increasing number of the minor G-allele. Bold horizontal line indicates the median, upper horizontal line indicates the upper 25% quartile, lower horizontal line indicates the lower 25% quartile

Furthermore, we have to note, that if the two samples were investigated separately, the association of SNP rs3852144 with negative pictures reached significance only in the fMRI sample (*b* = 0.08, *t*(1116) = 2.61, *p* = .009). Figure 3 displays the results, indicating a decrease of memory for negative pictures with an increase of the minor G-allele, which is consistent with the findings from the lifetime PTSD risk studies.

The effects for positive (*b* = 0.06, *t*(1116) = 1.93, *p* = .054) and for neutral pictures did not reach significance (*b* = 0.03, *t*(1116) = 0.95, *p* = .345) in the fMRI sample. None of the effects reached significance if only the EEG sample was included in the analyses, even though the direction of the effects was similar (negative: *b* = 0.02, *t*(1578) = 0.55, *p* = .584; positive: *b* = 0.01, *t*(1578) = 0.27, *p* = .786; neutral: *b* = 0.02, *t*(1578) = 0.61, *p* = .543). The interaction of genotype × sample was not significant, independent of the outcome variable used (*p* > .10).

### Ugandan therapy sample

Finally, we investigated whether SNP rs3852144 had an effect on the decrease of PTSD symptoms by exposure-based psychotherapy, including the PDS sum score as outcome variable, a time × genotype interaction as predictor and traumatic load and genotyping-batch as covariates. Statistical significance was confirmed by means of permutation tests (*p*_emp_). We found significant main effects for the factor time (*F*_2,174_ = 88.77, *p* < .001, *p*_emp_ < .001) and for traumatic load (*F*_1,85_ = 17.94, *p* < .001, *p*_emp_ < .001). Furthermore, a significant interaction between time × genotype on current PTSD symptom severity was observed (*F*_2,174_ = 4.15, *p* = .017, *p*_emp_ = .018), indicating that genotype influenced the symptom change over time. No significant main effects of genotyping-batch (*F*_2,85_ = 1.01, *p* = .370, *p*_emp_ = .391) or genotype (*F*_1,85_ = 0.40, *p* = .529, *p*_emp_ = .546) were found. To further evaluate the significant interaction, we calculated three planned general linear hypotheses adjusted for multiple comparisons. These post-hoc analyses revealed a significant effect of genotype on the change in PDS symptom score from pre-treatment to the 4-months follow-up (comparison t_1_–t_2_, *Z* = 2.33, *p*_adj_ = .029). Additionally, the symptom change between 4 months and 10 months after the end of therapy was associated with genotype (comparison t_2_–t_3_, *Z* = -2.18, *p*_adj_ = .042). However, the change in PTSD symptom severity from before therapy to the 10-months follow-up assessment (comparison t_1_–t_3_, *Z* = 0.15, *p*_adj_ = .885) was independent of genotype.

Descriptively, we can see that this effect seems to depend mainly on the homozygous G-allele carriers (see Supplementary Figure 8 and also Supplementary Tables 5 and 6). No pre-treatment differences between genotype groups in demographic and clinical data existed (for more details see Supplementary Table 6).

## Discussion

Our GWAS, performed in a sample of *N* = 924 Northern Ugandan rebel war survivors all exposed to the same large-scale conflict, resulted in seven SNPs passing the suggestive significance threshold of *p* ≤ 1 × 10^−5^ for associations with lifetime PTSD risk. For all SNPs, the individual effect was similarly found after application of a more stringent threshold to account for the high proportion of related individuals in the Ugandan sample. Furthermore, a highly significant main effect of the environmental factor traumatic load was found. However, only for SNP rs3852144 could the genetic association be replicated in an independent sample of Rwandan genocide survivors, indicating a decrease in lifetime PTSD risk with higher number of minor G-alleles. None of the SNPs replicated in the publicly available GWAS summary statistics of African Americans provided by the Psychiatric Genomics Consortium, which might be because of the different genetic background (East Africans versus African Americans) and the fact that in their GWAS the environmental factor traumatic load was not considered. Still, four of seven SNPs indicated a similar direction of effect. Investigations of rs3852144 in a healthy European sample indicated lower memory for pictures with negative valence with increasing number of the minor G-allele.

The effect of SNP rs3852144 on psychotherapy remains preliminary. Post-hoc investigations of the significant time × genotype interaction revealed a significant genotype effect on the symptom change from pre-treatment to four months after therapy, while no significant influence of genotype was observed regarding the symptom change from pre-treatment to ten months after treatment completion. Descriptively we can see that homozygous G-allele carriers showed higher symptom scores four months after therapy than heterozygotes and non-carriers. One might speculate that SNP rs3852144 G-allele is associated with diminished emotional memory formation, which leads to a reduced PTSD vulnerability, but on the other hand complicates memory extinction throughout the therapeutic progress. However, given the fact that the G/G-group consisted of only 10 individuals in the therapy sample, future analyses with larger sample size are needed to determine the consistency of the reported results and the putative biological impact of SNP rs3852144 on psychotherapy success.

According to the UCSC Human Genome Browser (GRCh37/hg19)^66^, SNP rs3852144 is located in the first intron of Chromosome 5 Open Reading Frame 67 (C5orf67), a protein-coding and brain-expressed gene with yet uncharacterized function. The SNP is surrounded by multiple conserved transcription factor binding sites involved in transcriptional regulation processes, the closest being V$OCT1_04, a binding target for transcription factor POU2F1, located 5 kb downstream. Furthermore, about 204 kb downstream of rs3852144, two long intervening noncoding RNAs (lincRNAs), TCONS_0009404 and TCONS_12_00022912, are localized^66^. The closest gene to rs3852144 is the Mitogen-Activated Protein Kinase Kinase Kinase 1 gene (*MAP3K1*) located 248 kb downstream^66^. MAP3K signaling via downstream targets is involved in inflammatory processes and in learning and memory formation (e.g.,^67^). However, the localization of SNP rs3852144 and the lack of biological data related to the open reading frame C5orf67 do not yet allow to draw any conclusions regarding the biological mechanisms that link this genetic variant to PTSD risk.

### Strengths and limitations

Strengths of the study include a systematic assessment of PTSD and traumatic load via structured clinical interviews in the African samples, each including individuals who survived the same conflict, and the inclusion of four different samples and three different phenotypic outcomes (i.e., PTSD risk, aversive memory performance and therapy responsiveness). A major limitation consists in the fact that all SNPs identified in our initial GWAS only reached suggestive significance, while none of them survived FDR correction for multiple testing. Thus, our results once again demonstrate the difficulties of GWAS to gain sufficient statistical power to detect minor genetic effect sizes in smaller samples, even if the study participants of each cohort were exposed to the same conflict and PTSD was assessed with the same measures. Nevertheless, our study represents a valuable contribution to the existing body of research in the field, demonstrating the potential to follow up suggestively significant GWAS results. Furthermore, our results again highlight the importance of the environmental factor traumatic load in terms of PTSD risk and treatment.

However, other factors known to increase the vulnerability for anxiety disorders but not included in this study (e.g., experience of childhood maltreatment^68^) should be accounted for in future research. Finally, although the size of the therapy sample was large for a treatment study, it was relatively small for a study on the genetics of treatment outcome. Only 10 individuals in the therapy sample presented with a current diagnosis of PTSD and the G/G-genotype that was previously associated with reduced PTSD vulnerability. Therefore, the results obtained from the therapy sample remain preliminary.

### Conclusions and further directions

In sum, the results give preliminary evidence for an association of SNP rs3852144 with PTSD risk and emotional memory indicating that this genetic variation might play a role in the consolidation of highly emotional memories after traumatic stress. Our findings underscore the pivotal role of memory processes in the aetiology of PTSD, as formulated in prominent neurocognitive models of PTSD development^34–40^. It further extends the growing literature suggesting a substantial overlap regarding genetic factors associated with memory performance in healthy individuals and the development and modification of trauma memories in PTSD.

Yet, the underlying biological processes mediating the observed association between SNP rs3852144 and emotional and traumatic memories still need to be clarified. In particular, future studies investigating the potential functional relationship of this intronic SNP with PTSD symptom development are needed. Studies implementing SNP imputation might also help to fine-map linked genetic variants, that are biologically more relevant and may underlie the observed associations. Furthermore, the potential role of rs3852144 in the modification of trauma memories by psychotherapy remains to be further investigated.

### Code availability

The PLINK code used to perform our GWAS and the R code used for all other calculations can be obtained from the Supplement.

See Supplementary Table 2 for an overview of the statistical tests conducted in each sample and the variables included. Information about the localization of significantly associated markers was obtained from the UCSC Human Genome Browser (GRCH37/hg19)^66^.

## Electronic supplementary material

Supplement clean version

## Data Availability

Requests to access the de-identified summary statistics of our genome-wide association analysis can be directed to iris.kolassa@uni-ulm.de.
