# Supplementary material for: Genetic variation is associated with PTSD risk and aversive memory: Evidence from two trauma-Exposed African samples and one healthy European sample
Source: Transl Psychiatry. 2018 Nov 22;8:251. doi: 10.1038/s41398-018-0297-1 (PMC6250662; doi:10.1038/s41398-018-0297-1)
Supplement: Supplementary file 1 — Supplement clean version [file 41398_2018_297_MOESM1_ESM.docx]

**Supplement**

1. **Supplementary Methods**

**Ugandan discovery and Rwandan replication sample: Detailed description of the recruitment procedure**

**Ugandan discovery sample.** For the study introduction, community meetings were set in which the purpose of the research project was explained. In the larger former internally displaced people (IDP) camps Anaka, Pabbo and Koch Goma, individuals were approached by the interviewers in their home stalls and where asked whether they were interested in study participation. In the smaller villages of Gulu district, where the huts are often scattered over far distance, interested individuals were invited to schedule an appointment for the diagnostic interview. In order to avoid a confounding of association results due to a sampling of related individuals (cryptic relatedness), only one member per household was allowed to participate in the study.

**Rwandan replication sample.** Participants were sampled proportionally to the population size of the zones of the refugee settlement in Nakivale, Uganda. Individuals were approached in their households and detailed information about the study was given. The member of each family who was most affected by the Rwandan genocide was invited to participate in the study.

**Ugandan discovery, Rwandan replication sample and healthy Swiss sample: Detailed description of the applied quality controls**

**Ugandan discovery sample.** The discovery sample comprised *N* = 1148 individuals. Subjects were excluded based on the following reasons, whereby some individuals were dropped for fulfilling multiple exclusion criteria: (1) inconsistency between reported sex and sex inferred from genoytpic data (*N* = 5) (2) genome-wide missing rate > 5% (*N* = 5) (3) subjects deviating in heterozygosity and missing rates, identified using Bayesian clustering (Bellenguez et al., 2012) (*N =* 15) (4) subjects with unusual ancestry genetic background according to the majority of the samples, identified using Bayesian clustering (Bellenguez et al., 2012) applied on the two first principal components inferred from HapMap CEU, YRI, CHB-JPT populations (*N =* 5) (5) subjects with cryptic relatedness (pi hat > 0.2) (one sample excluded for each pair, *N =* 180). Furthermore, stringent SNP quality criteria (minor allele frequency (MAF) > .05, SNP call rate > .95, non-deviance from Hardy-Weinberg equilibrium (HWE) > 0.05) were performed with PLINK software version 1.07 (Purcell et al., 2007) and lead to the exclusion of 239475 SNPs in the Ugandan discovery sample. Furthermore, only autosomal SNPs were considered, hence, analyses were based on 654099 autosomal SNPs.

**Rwandan replication sample.** The replication sample included *N =* 409 individuals. Subjects were excluded based on the following reasons, whereby some individuals were dropped for fulfilling multiple exclusion criteria: (1) inconsistency between reported sex and sex inferred from genoytpic data (*N =* 6) (2) missing rate > 5% (*N =* 1) (3) subjects deviating in heterozygosity and missing rates, identified using Bayesian clustering (Bellenguez et al., 2012) (*N =* 4) (4) subjects with unusual ancestry genetic background according to the majority of the samples, identified using Bayesian clustering (Bellenguez et al., 2012) applied on the two first principal components inferred from HapMap CEU, YRI, CHB-JPT populations (*N =* 6) (5) subjects with cryptic relatedness (pi hat > 0.2) (one sample excluded for each pair, *N =* 12). Furthermore, we assured that the SNP quality criteria applied to the Ugandan sample (i.e., MAF > .05, SNP call rate > .95, HWE > .05) were similarly fulfilled by all SNPs tested in the Rwandan sample.

**Healthy Swiss samples.** For each GWAS dataset, Bayesian Clustering Algorithm (Bellenguez et al., 2012) was applied on genome-wide summary statistics to identify and exclude atypical individual samples. Briefly, considering a combination of two summary statistics, the algorithm infers each sample's posterior probability to belong to the outliers' class. A first outlier assessment was based on genome-wide call rate and heterozygosis rates, for which extreme values may be indicative of a genotyping bias. The second assessment aimed at identifying subjects with unusual ancestry according to the majority of the samples. This was done by projecting the samples' genotypic data on the two first PCA components inferred from HapMap data using YRI, CEU and CHB-JPT populations. In addition, subjects with genome-wide missing rate > 5% were excluded. Further we performed a sex check between genetically inferred and self-reported gender and an IBD-check to exclude related subjects (pi hat > 0.2, one subject per identified pair excluded), within and between samples. Similar to the Ugandan and Rwandan sample, SNP quality controls were applied to all SNPs tested (i.e., MAF > .05, SNP call rate > .95, HWE > .05).

**PLINK and R codes**

#!/bin/bash

###########################################################################

### PLINK script to perform genome-wide association study in the Ugandan sample ###

### Sarah Wilker, Anna Schneider, Daniela Conrad ###

###########################################################################

### Notes

### 1. The home folder path needs to be replaced by your directory

### 2. Batch was dummy-coded for the analyses in PLINK

### 3. The phenotypic file contained 5 covariates all read in by the command "sex-Factor_batch2": sex, age, traumatic load, Factor_batch1, Factor_batch2

### 4. The following commands generates the files .log, .nosex, .assoc.logistic and .assoc.logistic.adjusted

### 5. The GWAS summary statistics for this study which are contained in the .assoc.logistic-file are available on request

./plink --noweb \

--bfile bed_bim_fam_file_name \

--out output_file_name \

--covar phenodata_file_name.txt \

--covar-name sex-Factor_batch2 \

--pheno phenodata_file_name.txt \

--pheno-name PTSD_LIFE_DSMIV \

--logistic \

--ci 0.95 \

--adjust

rm(list=ls())

#############################################################################################

### R script to perform single SNP analyses in the Ugandan sample ###

### Single SNP analyses included genetic variants that reached suggestive significance in the previous GWAS ###

### This script provides the code exemplary for SNP rs3852144 ###

### Sarah Wilker, Anna Schneider, Daniela Conrad ###

#############################################################################################

### Load necessary packages

library(car)

library(GenABEL)

library(psych)

#####################

### Read in data files ###

#####################

### Read in SNP files

### Note:

### Each SNP file contains the following information obtained from the UCSC genome browser:

### #bin, chrom, chromStart, chromEnd, name, score, strand, observed, rsId

SNP_chr5 <- as.character(read.table("chr5_sig_SNP.txt", sep="\t", comment.char="", header=TRUE)[,"rsId"])

### Read in previously generated GenABEL-object containing phenotypic and genetic data of your study sample

### Note:

### For the analyses applying a more stringent IBD threshold the same code was used, but a different GenABEL-object was read in containing less individuals.

load(file="discovery_data_file.Rdata")

### Select entries for SNPs found suggestively significant in previous GWAS

index <- rep(TRUE, nrow(phdata(discovery_data_file)))

gene <- discovery_data_file[index, SNP_chr5[SNP_chr5 %in% snpnames(discovery_data_file)]]

summary(gene)

#####################

### Data preparation ###

#####################

### Prepare dataset and check for missing values

### Note:

### The PTSD_LIFE_DSMIV-variable is binary coded: 0 = no lifetime PTSD diagnosis, 1 = lifetime PTSD diagnosis

### The SumEventTypesLife-variable is defined as the number of different traumatic event types experienced ("traumatic load")

data <- phdata(discovery_data_file)

data$rs3852144<- as.character(gene@gtdata[,"rs3852144"])

data$batch <- as.factor(data$batch)

data <- data [!is.na(data$PTSD_LIFE_DSMIV),]

data <- data [!is.na(data$SumEventTypesLife),]

### Test for single effect of covariates included in previous GWAS

### Note:

### Only SumEventTypesLife and batch reached significance

model_traumaload <- glm(PTSD_LIFE_DSMIV~SumEventTypesLife, data=data, family="binomial")

summary(model_traumaload)

Anova(model_traumaload, test = "LR")

model_batch <- glm(PTSD_LIFE_DSMIV~batch, data=data, family="binomial")

summary(model_batch)

Anova(model_batch, test = "LR")

model_sex <- glm(PTSD_LIFE_DSMIV~sex, data=data, family="binomial")

summary(model_sex)

Anova(model_sex, test = "LR")

model_age <- glm(PTSD_LIFE_DSMIV~age, data=data, family="binomial")

summary(model_age)

Anova(model_age, test = "LR")

### Prepare individual SNP data

data <- data [!is.na(data$rs3852144),]

summary(data$rs3852144)

### Generate new variable coding the genotype groups in an additive manner

data$additive_coding <- ifelse(data$rs3852144=="G/G", 2,

ifelse(data$rs3852144=="A/G", 1,

ifelse(data$rs3852144=="A/A", 0, data$rs3852144)))

###Check whether recoding worked out

cbind (data$rs3852144, data$additive_coding)

summary(data$additive_coding)

data$additive_coding <- as.numeric(data$additive_coding)

str(data$additive_coding)

###########################

### Single SNP analyses ###

###########################

### Test whether full or reduced model shows better fit and use model with smaller AIC subsequently

model.full <- glm(PTSD_LIFE_DSMIV~SumEventTypesLife*additive_coding+batch, data=data, family="binomial")

model.reduced <- glm(PTSD_LIFE_DSMIV~SumEventTypesLife+additive_coding+batch, data=data, family="binomial")

AIC(model.full)

AIC(model.reduced)

summary(model.reduced)

Anova(model.reduced, test = "LR")

### Note:

### Analysis of Deviance Table (Type II tests) contains the columns LR, Chisq Df, and Pr(>Chisq) reported in the manuscript

##########

### Plot ###

##########

xx <- seq(1, max(data$SumEventTypesLife, na.rm=TRUE))

newdata <- data.frame(SumEventTypesLife=rep(xx,3), additive_coding=rep(c(0, 1, 2), times=rep(max(xx), 3)))

newdata

model.reduced.plot <- glm(PTSD_LIFE_DSMIV~SumEventTypesLife+additive_coding, data=data, family="binomial")

prediction <- predict(model.reduced.plot, newdata=newdata, type="response")

tiff("Additive_model_rs3852144.tiff", width=3000, height=3000, res=400)

par(mai=c(2,1,1,1))

plot(xx, prediction[newdata$additive_coding==0], type="o", pch=21, bg="black", xlab="Number of Traumatic Event Types", cex.lab= 1.2, ylab="P(Lifetime PTSD)", ylim=c(0,1), main="Additive model rs3852144")

points(xx,prediction[newdata$additive_coding==1], type="o", pch=21, bg="grey")

points(xx,prediction[newdata$additive_coding==2], type="o", pch=21, bg="white")

legend(x="bottomright", pch=21, lty=1, pt.bg=c("black","grey", "white"), cex= 1.2, legend=c("A/A (N = 448)", "A/G (N = 388)", "G/G (N = 87)"))

dev.off()

################################

### Calculate Cronbach's Alpha ###

################################

alpha(data[,c("PDS1", "PDS2", "PDS3", "PDS4", "PDS5", "PDS6", "PDS7", "PDS8", "PDS9", "PDS10",

"PDS11", "PDS12", "PDS13", "PDS14", "PDS15", "PDS16", "PDS17")], check.keys=T)

###########

### END ###

###########

rm(list=ls())

###########################################################################

### R script to perform single SNP analyses in the Ugandan sample ###

### Replication attempt of single SNP analyses results in Rwandan genocide survivors ###

### This script provides the code exemplary for SNP rs3852144 ###

### Sarah Wilker, Anna Schneider, Daniela Conrad ###

###########################################################################

### Load necessary packages

library(car)

library(GenABEL)

library(psych)

#####################

### Read in data files ###

#####################

### Read in SNP files

### Note:

### Each SNP file contains the following information obtained from the UCSC genome browser:

### #bin, chrom, chromStart, chromEnd, name, score, strand, observed, rsId

SNP_chr5 <- as.character(read.table("chr5_sig_SNP.txt", sep="\t", comment.char="", header=TRUE)[,"rsId"])

### Read in previously generated GenABEL-object containing phenotypic and genetic data of your study sample

load(file="replication_data_file.Rdata")

### Select entries for SNP found suggestively significant in previous GWAS

index <- rep(TRUE, nrow(phdata(replication_data_file)))

gene <- replication_data_file[index, SNP_chr5[SNP_chr5 %in% snpnames(replication_data_file)]]

summary(gene)

#####################

### Data preparation ###

#####################

### Prepare dataset and check for missing values

### Note:

### Similar to the discovery_data_file the PTSD_LIFE_DSMIV-variable is binary coded: 0 = no lifetime PTSD diagnosis, 1 = lifetime PTSD diagnosis

### Similar to the discovery_data_file the SumEventTypesLife-variable is defined as the number of different traumatic event types experienced ("traumatic load")

data <- phdata(replication_data_file)

data$rs3852144 <- as.character(gene@gtdata[,"rs3852144"])

data <- data [!is.na(data$PTSD_LIFE_DSMIV),]

data <- data [!is.na(data$SumEventTypesLife),]

### Prepare individual SNP data

data <- data [!is.na(data$rs3852144),]

summary(data$rs3852144)

### Generate new variable coding the genotype groups in an additive manner (similar coding as for the discovery_data)

data$additive_coding <- ifelse(data$rs3852144=="G/G", 2,

ifelse(data$rs3852144=="A/G", 1,

ifelse(data$rs3852144=="A/A", 0, data$rs3852144)))

### Check whether recoding worked out

cbind (data$rs3852144, data$additive_coding)

summary(data$additive_coding)

data$additive_coding <- as.numeric(data$additive_coding)

str(data$additive_coding)

#########################################

### Replication of single SNP analyses results ###

#########################################

#### Define similar model as used for the discovery_data

model.reduced <- glm(PTSD_LIFE_DSMIV~SumEventTypesLife+additive_coding, data=data, family="binomial")

summary(model.reduced)

Anova(model.reduced, test = "LR")

### Note:

### Analysis of Deviance Table (Type II tests) contains the columns LR, Chisq Df and Pr(>Chisq) reported in the manuscript

###########

### Plot ###

###########

xx <- seq(1,max(data$SumEventTypesLife,na.rm=TRUE))

newdata <- data.frame(SumEventTypesLife=rep(xx,3), additive_coding=rep(c(0, 1, 2), times=rep(max(xx), 3)))

newdata

prediction <- predict(model.reduced,newdata=newdata, type="response")

par(mai=c(2,1,1,1))

plot(xx, prediction[newdata$additive_coding==0], type="o", pch=21, bg="black", xlab="Number of Traumatic Event Types", cex.lab= 1.2, ylab="P(Lifetime PTSD)", ylim=c(0,1), main="Additive model rs3852144")

points(xx, prediction[newdata$additive_coding==1], type="o", pch=21, bg="grey")

points(xx, prediction[newdata$additive_coding==2], type="o", pch=21, bg="white")

legend(x="bottomright", pch=21, lty=1, pt.bg=c("black","grey", "white"), cex= 1.2, legend=c("A/A (N = 202)", "A/G (N = 140)", "G/G (N = 28)"))

##############################

### Calculate Cronbach's Alpha ###

##############################

alpha(data[,c("PDS1", "PDS2", "PDS3", "PDS4", "PDS5", "PDS6", "PDS7", "PDS8", "PDS9", "PDS10",

"PDS11", "PDS12", "PDS13", "PDS14", "PDS15", "PDS16", "PDS17")], check.keys=T)

###########

### END ###

###########

rm(list=ls()); gc(); options(stringAsFactors=F)

###########################################################################

### R script to perform single SNP analyses in the Ugandan sample ###

### R script to test for associations of SNP rs3852144 (chr 5) with memory performance ###

### Sarah Wilker, Anna Schneider, Daniela Conrad ###

###########################################################################

#####################

### Read in data files ###

#####################

data_fMRI <- readRDS("data_fMRI_all_IAPS_ULM.rds")

data_EEG <- readRDS("data_EEG_all_IAPS_ULM.rds")

# Data dimensions prior to missing data exclusion

dim(data_EEG)

dim(data_fMRI)

#####################

### Data preparation ###

#####################

### Prepare dataset and check for missing values

### Note:

### The iaps_neu_day1_1-variable is defined as the short delay of picture set 1 at day 1

### 1. Phenotypic data

### Negative pictures

dim(data_EEG[!is.na(data_EEG$iaps_neg_day1_1),])

data_EEG_PHENOS <- data_EEG[!is.na(data_EEG$iaps_neg_day1_1),]

dim(data_fMRI[!is.na(data_fMRI$iaps_neg_day1_1),])

data_fMRI_PHENOS <- data_fMRI[!is.na(data_fMRI$iaps_neg_day1_1),]

### Neutral pictures

dim(data_EEG[!is.na(data_EEG$iaps_neu_day1_1),])

dim(data_fMRI[!is.na(data_fMRI$iaps_neu_day1_1),])

### Positive pictures

dim(data_EEG[!is.na(data_EEG$iaps_pos_day1_1),])

dim(data_fMRI[!is.na(data_fMRI$iaps_pos_day1_1),])

### 2. Genotypic data

dim(data_EEG_PHENOS[!is.na(data_EEG_PHENOS$rs3852144),])

data_EEG_GENOS_rs3852144 <- data_EEG_PHENOS[!is.na(data_EEG_PHENOS$rs3852144),]

dim(data_fMRI_PHENOS[!is.na(data_fMRI_PHENOS$rs3852144),])

data_fMRI_GENOS_rs3852144 <- data_fMRI_PHENOS[!is.na(data_fMRI_PHENOS$rs3852144),]

### Check data dimensions after exclusion

dim(data_EEG_GENOS_rs3852144)

dim(data_fMRI_GENOS_rs3852144)

### Regress out potential confounding effects and reduce datasets for later row-wise binding

### EEG data

data_EEG_FINAL <- data_EEG_GENOS_rs3852144

data_EEG_FINAL$myres_IAPS_POS_DAY_1 <- scale(lm(iaps_pos_day1_1 ~ factor(Sex) + Age + factor(Batch_Genetic), data= data_EEG_FINAL)$residuals)

data_EEG_FINAL$myres_IAPS_NEU_DAY_1 <- scale(lm(iaps_neu_day1_1 ~ factor(Sex) + Age + factor(Batch_Genetic), data= data_EEG_FINAL)$residuals)

data_EEG_FINAL$myres_IAPS_NEG_DAY_1 <- scale(lm(iaps_neg_day1_1 ~ factor(Sex) + Age + factor(Batch_Genetic), data= data_EEG_FINAL)$residuals)

data_EEG_FINAL$centre <- "EEG"

head(data_EEG_FINAL[1:20,])

EEG_FINAL <- data_EEG_FINAL[,c("Sex", "rs3852144", "myres_IAPS_NEG_DAY_1", "myres_IAPS_POS_DAY_1", "myres_IAPS_NEU_DAY_1", "centre")]

### fMRI data

data_fMRI_FINAL <- data_fMRI_GENOS_rs3852144

data_fMRI_FINAL$myres_IAPS_NEG_DAY_1 <- scale(lm(iaps_neg_day1_1 ~ factor(Sex) + Age + factor(Batch_Genetic) + factor(Batch_iaps_freerecall), data= data_fMRI_FINAL)$residuals)

data_fMRI_FINAL$myres_IAPS_POS_DAY_1 <- scale(lm(iaps_pos_day1_1 ~ factor(Sex) + Age + factor(Batch_Genetic) + factor(Batch_iaps_freerecall), data= data_fMRI_FINAL)$residuals)

data_fMRI_FINAL$myres_IAPS_NEU_DAY_1 <- scale(lm(iaps_neu_day1_1 ~ factor(Sex) + Age + factor(Batch_Genetic) + factor(Batch_iaps_freerecall), data= data_fMRI_FINAL)$residuals)

data_fMRI_FINAL$centre <- "fMRI"

head(data_fMRI_FINAL[1:20,])

fMRI_FINAL <- data_fMRI_FINAL[,c("Sex", "rs3852144", "myres_IAPS_NEG_DAY_1", "myres_IAPS_POS_DAY_1", "myres_IAPS_NEU_DAY_1", "centre")]

### rbind datasets

EEG_fMRI <- rbind(EEG_FINAL, fMRI_FINAL)

head(EEG_fMRI)

table(EEG_fMRI$Sex)

table(EEG_fMRI$rs3852144)

table(EEG_fMRI$centre)

#################################

### Regression analyses for ###

### EEG + fMRI cohort combined ###

#################################

### Make sure that genotype coding is the same as for the discovery_data and replication_data

table(EEG_fMRI$rs3852144)

str(EEG_fMRI$rs3852144)

### Negative pictures

model_neg_combined <- glm(myres_IAPS_NEG_DAY_1 ~ scale(rs3852144), data=EEG_fMRI, family="gaussian")

summary(model_neg_combined)

### Note: The summary statistics contain the columns Estimate, Std. Error, t value and Pr(>|t|) reported in the manuscript

### Positive pictures

model_pos_combined <- glm(myres_IAPS_POS_DAY_1 ~ scale(rs3852144), data=EEG_fMRI, family="gaussian")

summary(model_pos_combined)

### Neutral pictures

model_neu_combined <- glm(myres_IAPS_NEU_DAY_1 ~ scale(rs3852144), data=EEG_fMRI, family="gaussian")

summary(model_neu_combined)

###########

### Plot ###

###########

table(EEG_fMRI$rs3852144)

library(shape); library(beeswarm)

tiff("beeswarm_neg_day_1_1_EEG_fMRI.tiff", width=10000, height=3000, res=400)

par(mar = c(7,10,4,2) + 0.1, mgp=c(4,1,0))

beeswarm(myres_IAPS_NEG_DAY_1 ~ rs3852144, data=EEG_fMRI, main="Beeswarm Plot", labels=c("G/G (N = 372)", "A/G (N = 1283)", "A/A (N = 1043)"), xlab=" \n Genotype groups", ylab=" \n Recalled negative pictures (z-standardized)", method="swarm", cex.main=2, cex.lab=2, cex.axis=2)

bxplot(myres_IAPS_NEG_DAY_1 ~ rs3852144, data=EEG_fMRI, family="gaussian", add = TRUE)

dev.off

### Check for centre effects

model_centre_neg <- glm(myres_IAPS_NEG_DAY_1 ~ rs3852144*centre, data=EEG_fMRI, family="gaussian")

summary(model_centre_neg)

model_centre_pos <- glm(myres_IAPS_POS_DAY_1 ~ rs3852144*centre, data=EEG_fMRI, family="gaussian")

summary(model_centre_pos)

model_centre_neu <- glm(myres_IAPS_NEU_DAY_1 ~ rs3852144*centre, data=EEG_fMRI, family="gaussian")

summary(model_centre_neu)

###########################

### Regression analyses for ###

### EEG cohort only ###

###########################

### Negative pictures

model_neg_EEG <- glm(myres_IAPS_NEG_DAY_1 ~ rs3852144, data=EEG_fMRI[EEG_fMRI$centre=="EEG",], family="gaussian")

summary(model_neg_EEG)

### Positive pictures

model_pos_EEG <- glm(myres_IAPS_POS_DAY_1 ~ rs3852144, data=EEG_fMRI[EEG_fMRI$centre=="EEG",], family="gaussian")

summary(model_pos_EEG)

### Neutrale pictures

model_neu_EEG <- glm(myres_IAPS_NEU_DAY_1 ~ rs3852144, data=EEG_fMRI[EEG_fMRI$centre=="EEG",], family="gaussian")

summary(model_neu_EEG)

###########################

### Regression analyses for ###

### fMRI cohort only ###

###########################

### Negative pictures

model_neg_fMRI <- glm(myres_IAPS_NEG_DAY_1 ~ scale(rs3852144), data=EEG_fMRI[EEG_fMRI$centre=="fMRI",], family="gaussian")

summary(model_neg_fMRI)

### Positive pictures

model_pos_fMRI <- glm(myres_IAPS_POS_DAY_1 ~ scale(rs3852144), data=EEG_fMRI[EEG_fMRI$centre=="fMRI",], family="gaussian")

summary(model_pos_fMRI)

### Neutral pictures

model_neu_fMRI <- glm(myres_IAPS_NEU_DAY_1 ~ scale(rs3852144), data=EEG_fMRI[EEG_fMRI$centre=="fMRI",], family="gaussian")

summary(model_neu_fMRI)

###########

### Plot ###

###########

table(EEG_fMRI[EEG_fMRI$centre=="fMRI",]$rs3852144)

tiff("/Users/danielaconrad/Desktop/beeswarm_neg_day_1_fMRI.tiff", width=10000, height=5000, res=500)

par(mar = c(7,10,4,2) + 0.1, mgp=c(4,1,0))

beeswarm(myres_IAPS_NEG_DAY_1 ~ rs3852144, data=EEG_fMRI[EEG_fMRI$centre=="fMRI",], main="Beeswarm Plot", labels=c("G/G (N = 159)", "A/G (N = 537)", "A/A (N = 422)"), xlab=" \n Genotype groups", ylab=" \n Recalled negative pictures (z-standardized)", method="swarm", cex.main=2, cex.lab=2, cex.axis=2)

bxplot(myres_IAPS_NEG_DAY_1 ~ rs3852144, data=EEG_fMRI[EEG_fMRI$centre=="fMRI",], family="gaussian", add = TRUE)

dev.off

###########

### END ###

###########

rm(list=ls())

###########################################################################

### R script to perform single SNP analyses in the Ugandan sample ###

### R script to test for associations of SNP rs3852144 (chr 5) with therapy response ###

### Sarah Wilker, Anna Schneider, Daniela Conrad ###

###########################################################################

### Load necessary packages

library(GenABEL)

library(nlme)

library(sciplot)

library(multcomp)

library(phia)

library(effects)

library(lsmeans)

library(AICcmodavg)

library(ggplot2)

library(effsize)

library(psych)

#####################

### Read in data files ###

#####################

### Read in SNP files

### Note:

### Each SNP file contains the following information obtained from the UCSC genome browser:

### #bin, chrom, chromStart, chromEnd, name, score, strand, observed, rsId

SNP_chr5 <- as.character(read.table("chr5_sig_SNP.txt",sep="\t", comment.char="", header=TRUE)[,"rsId"])

### Read in previously generated GenABEL-object containing phenotypic and genetic data of your study sample

load(file="therapy_data_file.Rdata")

### Select entries for SNPs found suggestively significant in previous GWAS

index <- rep(TRUE,nrow(phdata(therapy_data_file))) # alle VPs

gene <- therapy_data_file[index,SNP_chr5[SNP_chr5 %in% snpnames(therapy_data_file)]]

summary(gene)

#####################

### Data preparation ###

#####################

### Prepare dataset and check for missing values

### Note:

### Similar to the discovery_data_file the SumEventTypesLife-variable is defined as the number of different traumatic event types experienced ("traumatic load")

data <- phdata(therapy_data_file)

data$rs3852144 <- as.character(gene@gtdata[,"rs3852144"])

data$batch <- as.factor(data$batch)

data <- data [!is.na(data$SumEventTypesLife_t1),] # none

### Note:

### Missing values in the dependent variable are not problematic in lme-models as long as not the same individuals presents with more than one missing value

data_missing_t1 <- data [!is.na(data$SUM_PDS_t1),]

data_missing_t2 <- data [!is.na(data$SUM_PDS_t2),]

data_missing_t3 <- data [!is.na(data$SUM_PDS_t3),]

### Prepare individual SNP data

data <- data [!is.na(data$rs3852144),]

summary(data$rs3852144)

### Generate new variable coding the genotype groups in an additive manner (similar coding as for the discovery_data and replication_data)

data$additive_coding <- ifelse(data$rs3852144=="G/G",2,

ifelse(data$rs3852144=="A/G",1,

ifelse(data$rs3852144=="A/A",0,data$rs3852144)))

###Check whether recoding worked out

cbind (data$rs3852144, data$additive_coding)

summary(data$additive_coding)

data$additive_coding <- as.numeric(data$additive_coding)

str(data$additive_coding)

##### Restructuring data for lme calculations

data.long.chr5 <- reshape (data,direction = "long",

varying =c("SUM_PDS_t1", "SUM_PDS_t2", "SUM_PDS_t3" ),

idvar="index_Therapystudy", ids="index_Therapystudy",

v.names="PDS_Score", times=c("t1", "t2", "t3"))

data.long.chr5$time <- as.factor(data.long.chr5$time)

data.long.chr5$sex <- as.factor(data.long.chr5$sex)

##################################

##### Linear mixed effect model ####

##################################

#######################

### Model comparisons ###

#######################

### Before testing the model, test whether random slope improves model fit

model.random.intercept <- lme(PDS_Score ~ time, random=~1|index_Therapystudy,

data=data.long.chr5, na.action=na.omit, method="ML")

model.random.slope <- lme(PDS_Score~time, random=~time|index_Therapystudy,

data=data.long.chr5, na.action=na.omit, method="ML", control = lmeControl(opt = "optim"))

anova(model.random.intercept, model.random.slope)

#### Test for associations of SNP rs3852144 with lifetime PTSD risk including the same covariates as in the GWAS and single SNP analyses

model <- lme (PDS_Score~time*additive_coding+SumEventTypesLife+batch,

random=~1|index_Therapystudy, data=data.long.chr5,

na.action=na.omit, method="ML",

correlation=corSymm(form=~1|index_Therapystudy))

summary(model)

anova(model, type="marginal")

# Note:

# Analysis of Deviance Table contains columns numDF, denDF, F-value, and p-value reported in the manuscript

#####################################################################

### Planned linear hypothesis to follow-up significant time*genotype interaction ###

#####################################################################

K_add_interaction <- rbind (c(0,0,0,0,0,0,0,1,0),

c(0,0,0,0,0,0,0,0,1),

c(0,0,0,0,0,0,0,-1,1))

dimnames(K_add_interaction) <- list(c("add: t1-t2",

"add : t1-t3",

"add : t2-t3"),

names(fixef(model)))

summary(glht(model,linfct=K_add_interaction), test=adjusted("holm"))

#####################################################################

### Perform permutation tests as model residuals are non-normally distributed ###

### Exemplary described for the interaction effect, but done for all effects ###

#####################################################################

shapiro.test(residuals(model))

### Permutation Interaction effect

p.old <- .0173

n.perm <- 10000

perm.test <- data.frame(matrix(NA,n.perm,ncol=0))

for (ii in 1:n.perm) {

set.seed(ii)

PDS_Score.new <- sample (data.long.chr5$PDS_Score)

model.new <- lme (PDS_Score.new~time*additive_coding+SumEventTypesLife_t1+batch,

random=~1|index_Therapystudy, data=data.long.chr5,

na.action=na.omit, method="ML",

correlation=corSymm(form=~1|index_Therapystudy))

anova <- anova(model.new)

perm.test$pvalue.anova.new[ii] <- anova [6,4]

}

p.perm <- sum (perm.test$pvalue.anova.new <= p.old)/n.perm

p.perm ### 0.0182

###########

### Plot ###

###########

par(mai=c(2,1,1,1))

lineplot.CI(x.factor=data.long.chr5$time, response=data.long.chr5$PDS_Score, group=data.long.chr5$additive_coding,lwd=2,legend=TRUE,

xlab="Time", ylab="PDS Score", ylim = c(0, 20), col = c("black","grey20", "grey66"), lty = c(1,2,2), pch = c(16,16,16), cex=1.2, main="Additive model rs3852144")

abline (a=0, b=0, col= "gray60")

abline (a=2.5, b=0, col= "gray60")

abline (a=5, b=0, col= "gray60")

abline (a=7.5, b=0, col= "gray60")

abline (a=10, b=0,col= "gray60")

abline (a=12.5, b=0, col= "gray60")

abline (a=15, b=0, col= "gray60")

abline (a=17.5, b=0, col= "gray60")

abline (a=20, b=0, col= "gray60")

legend(x="topright",bg="white",col = c("grey66","grey20","black"), lty = c(2,2,1), pch = c(16,16,16),legend=c("G/G (N = 10)","A/G (N = 36)","A/A (N = 44)"),title = "rs3852144 Genotypes")

##########################

### Supplementary Table 5 ###

##########################

###PTSD diagnosis

sum(data[data$additive_coding==2,]$PTSD_current_DSMIV_yn_t1,na.rm=T)

sum(data[data$additive_coding==2,]$PTSD_current_DSMIV_yn_t2,na.rm=T)

sum(data[data$additive_coding==2,]$PTSD_current_DSMIV_yn_t3,na.rm=T)

sum(data[data$additive_coding==1,]$PTSD_current_DSMIV_yn_t1,na.rm=T)

sum(data[data$additive_coding==1,]$PTSD_current_DSMIV_yn_t2,na.rm=T)

sum(data[data$additive_coding==1,]$PTSD_current_DSMIV_yn_t3,na.rm=T)

sum(data[data$additive_coding==0,]$PTSD_current_DSMIV_yn_t1,na.rm=T)

sum(data[data$additive_coding==0,]$PTSD_current_DSMIV_yn_t2,na.rm=T)

sum(data[data$additive_coding==0,]$PTSD_current_DSMIV_yn_t3,na.rm=T)

#####################################################################

### Supplementary Table 6 ###

### Comparison of PDS-symptom scores between genotypes at t1, t2 and t3 ###

### and calculation of effect sizes of symptom change ###

#####################################################################

my.variables <- data[,c("SUM_PDS_t1", "SUM_PDS_t2", "SUM_PDS_t3")]

table.means <- data.frame(matrix(NA,length(my.variables),ncol=0))

sig.table <- data.frame(matrix(NA,length(my.variables),ncol=0))

for (ii in 1:ncol(my.variables)) {

table.means$variable[ii] <- names(my.variables[ii])

table.means$mean_sd_G_G[ii] <- paste (round(mean(my.variables[data$additive_coding == 2,ii] , na.rm=T),2), " (",

round(sd(my.variables[data$additive_coding == 2,ii] , na.rm=T),2), ")", sep="")

table.means$mean_sd_A_G[ii] <- paste (round(mean(my.variables[data$additive_coding == 1,ii] , na.rm=T),2), " (",

round(sd(my.variables[data$additive_coding == 1,ii] , na.rm=T),2), ")", sep="")

table.means$mean_sd_A_A[ii] <- paste (round(mean(my.variables[data$additive_coding == 0,ii] , na.rm=T),2), " (",

round(sd(my.variables[data$additive_coding == 0,ii] , na.rm=T),2), ")", sep="")

}

###########

### t1:t3 ###

###########

data$change_t1_t3 <- data$SUM_PDS_t1-data$SUM_PDS_t3

summary(data)

### G/G genotype

mean(data[data$additive_coding == 2,]$change_t1_t3)

round(sd(data[data$additive_coding == 2,]$change_t1_t3), 2)

effect <- cohen.d(data[data$additive_coding==2,]$SUM_PDS_t1,data[data$additive_coding==2,]$SUM_PDS_t3)

round(effect$estimate,2)

### A/G genotype

mean(data[data$additive_coding == 1,]$change_t1_t3)

round(sd(data[data$additive_coding == 1,]$change_t1_t3), 2)

effect <- cohen.d(data[data$additive_coding==1,]$SUM_PDS_t1,data[data$additive_coding==1,]$SUM_PDS_t3)

round(effect$estimate,2)

### A/A genotype

mean(data[data$additive_coding == 0,]$change_t1_t3, na.rm=T)

round(sd(data[data$additive_coding == 0,]$change_t1_t3, na.rm = T), 2)

effect <- cohen.d(data[data$additive_coding==0,]$SUM_PDS_t1,data[data$additive_coding==0,]$SUM_PDS_t3,na.rm = T)

round(effect$estimate,2)

###########

### t1:t2 ###

###########

data$change_t1_t2 <- data$SUM_PDS_t1-data$SUM_PDS_t2

summary(data)

### G/G genotype

mean(data[data$additive_coding == 2,]$change_t1_t2)

round(sd(data[data$additive_coding == 2,]$change_t1_t2), 2)

effect <- cohen.d(data[data$additive_coding==2,]$SUM_PDS_t1,data[data$additive_coding==2,]$SUM_PDS_t2)

round(effect$estimate,2)

### A/G genotype

mean(data[data$additive_coding == 1,]$change_t1_t2)

round(sd(data[data$additive_coding == 1,]$change_t1_t2), 2)

effect <- cohen.d(data[data$additive_coding==1,]$SUM_PDS_t1,data[data$additive_coding==1,]$SUM_PDS_t2)

round(effect$estimate,2)

### A/A genotype

mean(data[data$additive_coding == 0,]$change_t1_t2, na.rm=T)

round(sd(data[data$additive_coding == 0,]$change_t1_t2, na.rm = T), 2)

effect <- cohen.d(data[data$additive_coding==0,]$SUM_PDS_t1,data[data$additive_coding==0,]$SUM_PDS_t2,na.rm = T)

round(effect$estimate,2)

###########

### t2:t3 ###

###########

data$change_t2_t3 <- data$SUM_PDS_t2-data$SUM_PDS_t3

summary(data)

### G/G genotype

mean(data[data$additive_coding == 2,]$change_t2_t3)

round(sd(data[data$additive_coding == 2,]$change_t2_t3), 2)

effect <- cohen.d(data[data$additive_coding==2,]$SUM_PDS_t2,data[data$additive_coding==2,]$SUM_PDS_t3)

round(effect$estimate,2)

### A/G genotype

mean(data[data$additive_coding == 1,]$change_t2_t3)

round(sd(data[data$additive_coding == 1,]$change_t2_t3), 2)

effect <- cohen.d(data[data$additive_coding==1,]$SUM_PDS_t2,data[data$additive_coding==1,]$SUM_PDS_t3)

round(effect$estimate,2)

### A/A genotype

mean(data[data$additive_coding == 0,]$change_t2_t3, na.rm=T)

round(sd(data[data$additive_coding == 0,]$change_t2_t3, na.rm = T), 2)

effect <- cohen.d(data[data$additive_coding==0,]$SUM_PDS_t2,data[data$additive_coding==0,]$SUM_PDS_t3,na.rm = T)

round(effect$estimate,2)

###########################################################

### Supplementary Table 7 ###

### Test for differences in variable means other than genotype at t1 ###

### to exclude their influence on therapy response ###

###########################################################

names(data)

my.variables <- data[,c("age_t1","SumEventTypesLife_t1",

"SUM_PDS_t1", "SUM_PDS_I_t1",

"SUM_PDS_A_t1", "SUM_PDS_H_t1",

"HSCL_D_t1")]

my.variables$HSCL_D_t1 <- as.numeric(gsub("," , ".", data$HSCL_D_t1))

table <- data.frame(matrix(NA,length(my.variables),ncol=0))

for (ii in 1:nrow(table)) {

table$variable[ii] <- names(my.variables[ii])

table$mean_sd_G_G[ii] <- paste (round(mean(my.variables[data$additive_coding==2,ii] , na.rm=T),2), " (",

round(sd(my.variables[data$additive_coding==2,ii] , na.rm=T),2), ")", sep="")

table$mean_sd_A_G_Allele[ii] <- paste (round(mean(my.variables[data$additive_coding==1,ii] , na.rm=T),2), " (",

round(sd(my.variables[data$additive_coding==1,ii] , na.rm=T),2), ")", sep="")

table$mean_sd_A_A_Allele[ii] <- paste (round(mean(my.variables[data$additive_coding==0,ii] , na.rm=T),2), " (",

round(sd(my.variables[data$additive_coding==0,ii] , na.rm=T),2), ")", sep="")

my.test <- lm (my.variables [,ii]~additive_coding, data=data)

table$shapiro.p [ii] <- round(shapiro.test (residuals(my.test))$p.value,4)

table$shapiro.crit [ii] <- shapiro.test (residuals(my.test))$p.value < 0.05

anova <- round(unlist(summary(aov(my.variables[,ii]~data$additive_coding))),2)

table$anova[ii] <- paste ("F(",anova["Df1"],",",anova["Df2"],")=",anova["F value1"],"; p=",anova["Pr(>F)1"], sep="")

kruskal <- kruskal.test(my.variables[,ii]~data$additive_coding)

table$kruskal[ii] <- paste ("H(",kruskal["parameter"],")=",kruskal["statistic"],"; p=",kruskal["p.value"], sep="")

}

###Gender

sum(data[data$additive_coding==2,]$sex==0,na.rm=T)

sum(data[data$additive_coding==1,]$sex==0,na.rm=T)

sum(data[data$additive_coding==0,]$sex==0,na.rm=T)

fisher.test(data$sex,data$additive_coding)

##############################

### Calculate Cronbach's Alpha ###

##############################

alpha(data[,c("PDS1_t1", "PDS2_t1", "PDS3_t1", "PDS4_t1", "PDS5_t1", "PDS6_t1", "PDS7_t1", "PDS8_t1", "PDS9_t1", "PDS10_t1",

"PDS11_t1", "PDS12_t1", "PDS13_t1", "PDS14_t1", "PDS15_t1", "PDS16_t1", "PDS17_t1")], check.keys=T)

###########

### END ###

###########

1. **Supplementary results**

Supplementary Table 1.

*Overview of the 10 most frequently experienced events in the Ugandan discovery and Rwandan replication sample.*

|  | **Ugandan discovery sample (*N* = 924)** | |  | | **Rwandan replication sample (*N* = 371)** | | |  | **Ugandan therapy sample (*N* = 90)** | |
| --- | --- | --- | --- | --- | --- | --- | --- | --- | --- | --- |
| No. | Event description | Frequency of exposure (N, %) |  | Event description | | Frequency of exposure (N, %) |  | | Event description | Frequency of exposure (N, %) |
| 1. | Been close to a combat situation | 860, 93.07 |  | Seen mutilations or dead bodies | | 308, 83.02 |  | | Been close to a combat situation | 86, 95.56 |
| 2. | Witnessed beatings or torture | 844, 91.34 |  | Witness beatings or torture | | 297, 80.05 |  | | Been close to burning houses | 83, 92.22 |
| 3. | Witnessed friend or family member having life-threatening illness | 799, 86.47 |  | Witnessed someone being severely injured by weapon | | 281, 75.74 |  | | Witnessed beatings or torture | 82, 91.11 |
| 4. | Witnessed abduction or recruitment by force^1^ | 783, 84.74 |  | Been close to shelling or bomb attack | | 247, 66.58 |  | | Witnessed friend or family member having life-threatening illness | 80, 88.89 |
| 5. | Witnessed road accident | 767, 83.01 |  | Witness harassment by armed personnel | | 244, 65.77 |  | | Witnessed threat by weapon | 74, 82.22 |
| 6. | Been close to burning houses | 753, 81.49 |  | Been close to crossfire or shooting of snipers | | 234, 63.07 |  | | Witnessed road accident | 73, 81.11 |
| 7. | Witnessed threat by weapon | 751, 81.28 |  | Been close to burning houses | | 229, 61.73 |  | | Experienced unexpected death of someone close | 72, 80.00 |
| 8. | Witnessed someone being severely injured by weapon | 751, 81.28 |  | Been harassed by armed personnel | | 208, 56.06 |  | | Suffered from life-threatening illness or injury | 72, 80.00 |
| 9. | Been close to crossfire or shooting of snipers | 743, 80.41 |  | Witness the killing or murder of someone | | 199, 53.64 |  | | Witnessed other kind of severe accident (no road accidents) | 72, 80.00 |
| 10. | Been close to shelling or bomb attack | 743, 80.41 |  | Witness severe accident | | 199, 53.64 |  | | Been close to crossfire or shooting of snipers | 72, 80.00 |

Supplementary Table 2.

*Overview of statistical tests conducted in each sample and variables included.*

| **Sample** | **Statistical model** | **Dependent variable** | **Independent variable(s)** | **Covariates** |
| --- | --- | --- | --- | --- |
| **Ugandan discovery sample** | Logistic regression model | PTSD lifetime diagnosis | Genotype (additive effect) | Traumatic load  Sex  Age  Genotyping-batch |
| **Rwandan replication sample** | Logistic regression model | PTSD lifetime diagnosis | Genotype (additive effect) | Traumatic load |
| **Ugandan therapy sample** | Linear mixed effect model | PTSD symptom severity | Genotype (additive effect)  Time  Genotype (additive effect) × Time | Traumatic load  Genotyping-batch |
| **Healthy Swiss sample** | Linear regression model | Number of memorized negative/positive/neutral pictures | Genotype (additive effect) | none^1^ |

*Note:* PTSD = Posttraumatic Stress Disorder

^1^ To avoid potential confounding of the analyses of the healthy Swiss sample by age, sex and genotyping-batch and change of the environmental condition in the fMRI study, the respective effects were regressed out.

Supplementary Table 3.

*Detailed information on the SNPs separately tested in each sample.*

| **Sample** | **SNP** | **Non-missing data samples** | **Minor allele frequency**^a^ | **Hardy-Weinberg equilibrium**^b^ |
| --- | --- | --- | --- | --- |
| **Ugandan discovery sample** | chr 2, rs570877 | *N* = 924 | 0.21 | *p =* .59 |
|  | chr 3, rs6773270 | *N* = 915 | 0.23 | *p* = .92 |
|  | chr 3, rs6798512 | *N =* 921 | 0.23 | *p =* .97 |
|  | chr 5, rs3852144* | *N* = 923 | 0.30 | *p =* .82 |
|  | chr 5, rs7700424 | *N =* 916 | 0.39 | *p =* .74 |
|  | chr 6, rs2237110 | *N* = 915 | 0.31 | *p =* .16 |
|  | chr 13, rs2892713 | *N* = 911 | 0.12 | *p =* .86 |
| **Rwandan replication sample**^c^ | chr 2, rs570877 | *N* = 371 | 0.20 | *p =* .96 |
|  | chr 3, rs6773270 | *N* = 369 | 0.30 | *p* = .86 |
|  | chr 3, rs6798512 | *N =* 369 | 0.30 | *p* = .98 |
|  | chr 5, rs3852144* | *N* = 370 | 0.27 | *p =* .59 |
|  | chr 5, rs7700424 | *N =* 359 | 0.44 | *p =* .87 |
|  | chr 6, rs2237110 | *N* = 370 | 0.28 | *p =* .98 |
| **Ugandan therapy sample** | chr 5, rs3852144* | *N* = 90 | 0.31 | *p =* .53 |
| **Healthy Swiss sample** | chr 5, rs3852144* | *N* = 2698 | 0.38 | *p =* .47 |

*Note:* SNP = single nucleotide polymorphism

SNP rs3852144, which was associated with PTSD risk, therapy outcome and healthy memory is marked with an asterisk.

^a^ minor allele rs570877 = T; minor allele rs6773270 = G, minor allele rs6798512 = A, minor allele rs3852144 = G, minor allele rs7700424 = C, minor allele rs2237110 = T, minor allele rs2892713 = T; ^b^ Chi-squares test results; ^c^ SNP rs2892713 could not be tested for replication in the Rwandan sample as it did not meet the applied SNP quality control criteria in this cohort.

Supplementary Table 4.

*Comparison of results for suggestively significant GWAS SNPs with publicly available PTSD GWAS data*

| SNP | *Lifetime PTSD GWAS results from Northern Ugandan rebel war survivors* | | | | |  | *Lifetime PTSD GWAS results from publicly available African American cohort* | | | | |
| --- | --- | --- | --- | --- | --- | --- | --- | --- | --- | --- | --- |
|  | Allele 1 (effect allele) | Allele 2 | Effect | *Standard error* | *P-*value |  | Allele 1 (effect allele) | Allele 2 | Effect | *Standard error* | *P-*value |
| rs570877 (chr 2)* | T | G | -4.649 | 0.144 | 3.341×10^-06^ |  | T | G | 0.005 | 0.045 | .919 |
| rs6773270 (chr 3)* | G | A | 4.500 | 0.152 | 6.798×10^-06^ |  | A | G | 0.018 | 0.041 | .655 |
| rs6798512 (chr 3)* | A | G | 4.421 | 0.152 | 9.830×10^-06^ |  | A | G | -0.018 | 0.041 | .656 |
| rs3852144 (chr 5) | G | A | -4.596 | 0.125 | 4.316×10^-06^ |  | A | G | 0.011 | 0.037 | .771 |
| rs7700424 (chr 5) | C | T | 4.433 | 0.126 | 9.280×10^-06^ |  | T | C | -0.066 | 0.036 | .064 |
| rs2237110 (chr 6) | T | G | -4.561 | 0.124 | 5.092×10^-06^ |  | T | G | -0.061 | 0.047 | .192 |
| rs2892713 (chr 13) | T | C | -4.813 | 0.174 | 1.488×10^-06^ |  | T | C | -0.039 | 0.060 | .520 |

*Note*: The GWAS in the Northern Ugandan sample included traumatic load as a covariate, while the GWAS performed with the African American sample did not. For SNPs rs6773270, rs3852144 and rs7700424 the effect allele in the Ugandan cohort is opposite to the effect allele reported in the GWAS summary statistics provided by the Psychiatric Genomics Consortium. *Only SNPs rs570877, rs6773270 and rs6798512 indicate a contrary effect in the two cohorts.

Supplementary Table 5.

*Number of participants with current PTSD diagnosis before therapy (t1), 4 months after therapy (t2) and at 10 months follow-up (t3), separately displayed for genotype groups of rs3852144 in the Ugandan therapy sample.*

| **SNP** |  | **Genotype** | **PTSD diagnosed individuals t1** | **PTSD diagnosed individuals t2** | **PTSD diagnosed individuals t3** |
| --- | --- | --- | --- | --- | --- |
| **rs3852144** |  | G/G (*N* = 10) | 10 (100.00%) | 7 (70.00%) | 1 (10.00%) |
|  |  | A/G (*N* = 36) | 36 (100.00%) | 7 (19.44%) | 10 (27.78%) |
|  |  | A/A (*N* = 44) | 44 (100.00%) | 12 (27.27%) | 16 (36.36%) |

*Note:* SNP = single nucleotide polymorphism; PTSD = posttraumatic stress disorder

Supplementary Table 6.

*Mean and standard deviation of PDS scores before therapy (t1), 4-months after therapy (t2) and at 10-months follow up (t3).*

| **SNP** |  | **Genotype** | **Mean (s.d.) PDS score** | | | **Change Score**  **t1 - t2**^c^ | **Effect size Cohen’s D**  **t1 - t2**^d^ | **Change Score**  **t1 - t3**^c^ | **Effect size Cohen’s D**  **t1 - t3**^d^ | **Change Score**  **t2 - t3**^c^ | **Effect size Cohen’s D t2 - t3**^d^ |
| --- | --- | --- | --- | --- | --- | --- | --- | --- | --- | --- | --- |
|  |  |  |  | | |  |  |  |  |  |  |
|  |  |  | **t1** | **t2**^a^ | **t3**^b^ |  |  |  |  |  |  |
| **rs3852144** |  | G/G  (*N* = 10) | 15.80 (4.54) | 12.30 (6.18) | 7.20  (3.82) | - 3.50  (6.85) | 0.65 | - 8.60  (7.47) | 2.05 | - 5.10  (7.42) | 0.99 |
|  |  | A/G  (*N* = 36) | 16.69 (4.59) | 6.92 (5.26) | 7.08  (5.81) | - 9.78  (5.25) | 1.98 | - 9.61  (6.17) | 1.84 | + 0.17 (6.92) | 0.03 |
|  |  | A/A  (*N* = 44) | 17.16 (5.03) | 7.09 (4.56) | 7.84  (5.63) | - 10.23  (5.63) | 2.10 | - 9.26  (6.54) | 1.75 | + 0.71 (5.20) | 0.15 |

*Note:* SNP = single nucleotide polymorphism; PDS = Posttraumatic Stress Diagnostic Scale

^a^ One individual was not found for the post-test 4-months after therapy.

^b^ One individual was not found for 10-months follow-up.

^c^ Change score describes the averaged difference in within-group PDS sum scores between pre-treatment and 4-months follow up, pre-treatment and 10-months follow-up, and between 4- and 10-months follow up, respectively.

^d^ Cohen’s *D* describes the treatment effect size between pre-treatment and 4-months follow up, pre-treatment and 10-months follow-up, and between 4- and 10-months follow up assessment calculated separately for each genotype group.

Supplementary Table 7.

*Demographic overview of rs3852144 genotype groups in the Ugandan therapy sample.*

|  | **G/G**  **(*N* = 10)** | **A/G**  **(*N* = 36)** | **A/A**  **(*N* = 44)** | **Statistic^a^** | ***P*-value** |
| --- | --- | --- | --- | --- | --- |
| **N female (%)** | 7 (70) | 18 (50) | 30 (68) | Fisher’s exact test | p = .22 |
| **Mean age (s.d.)** | 33.30 (10.72) | 29.33 (7.27) | 32.52 (10.16) | H(2) = 2.06 | p = .36 |
| **Mean traumatic load (s.d.)** | 37.50 (5.5) | 36.28 (6.40) | 37.48 (7.00) | F(1,88) = 0.14 | p = .71 |
| **Mean number of sessions (s.d.)** | 11.9 (2.02) | 11.31 (1.51) | 12.27 (2.09) | H(2) = 1.25 | p = .54 |
| **Mean PDS score (t1) (s.d.)** | 15.8 (4.54) | 16.69 (4.59) | 17.16 (5.03) | H(2) = 0.59 | p = .74 |
| **Mean PDS intrusions score (t1) (s.d.)** | 3.90 (1.10) | 4.50 (2.08) | 5.02 (2.22) | F(1,88) = 2.92 | p = .09 |
| **Mean PDS avoidance score (t1) (s.d.)** | 5.90 (2.81) | 6.00 (1.82) | 5.91 (2.09) | H(2) = 0.73 | p = .70 |
| **Mean PDS hyperarousal score (t1) (s.d.)** | 6.00 (2.05) | 6.19 (2.48) | 6.23 (2.22) | H(2) = 0.10 | p = .95 |
| **Mean HSCL-D score (t1) (s.d.)** | 2.32 (0.70) | 2.32 (0.78) | 2.47 (0.74) | H(2) = 0.59 | p = .75 |

^a^ One-way analysis of variance for continuous data if test residuals were normally distributed according to Shapiro Wilk’s W test; Kruskal-Wallis H test for continuous, possibly tied data if residuals were not normally distributed; Fisher’s exact test for categorical data; PDS = Posttraumatic Stress Diagnostic Scale; HSCL-D = Hopkins Symptom Checklist for Depression.


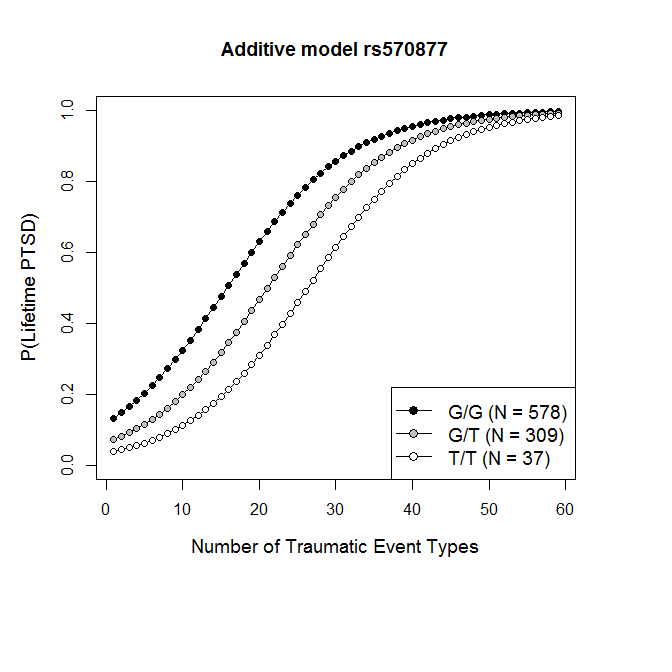


*Supplementary Figure 1.* *Ugandan discovery sample.* Fitted probability values for lifetime posttraumatic stress disorder (PTSD) as a function of traumatic load are plotted separately for the three genotype groups of rs570877 (chromosome 2). Results show a decreased risk for PTSD development after traumatic experiences with increasing number of the minor T-allele.

**

*Supplementary Figure 2.* *Ugandan discovery sample.* Fitted probability values for lifetime posttraumatic stress disorder (PTSD) as a function of traumatic load are plotted separately for the three genotype groups of rs2237110 (chromosome 6). Results show a decreased risk for PTSD development after traumatic experiences with increasing number of the minor T-allele.

*Supplementary Figure 3.* *Ugandan discovery sample.* Fitted probability values for lifetime posttraumatic stress disorder (PTSD) as a function of traumatic load are plotted separately for the three genotype groups of rs2892713 (chromosome 13). Results show a decreased risk for PTSD development after traumatic experiences with increasing number of the minor T-allele.

**

*Supplementary Figure 4.* *Ugandan discovery sample.* Fitted probability values for lifetime posttraumatic stress disorder (PTSD) as a function of traumatic load are plotted separately for the three genotype groups of rs6773270 (chromosome 3). Results indicate an increased risk for PTSD development after traumatic experiences with increasing number of the minor G-allele.

*Supplementary Figure 5.* *Ugandan discovery sample.* Fitted probability values for lifetime posttraumatic stress disorder (PTSD) as a function of traumatic load are plotted separately for the three genotype groups of rs6798512 (chromosome 3). Results indicate an increased risk for PTSD development after traumatic experiences with increasing number of the minor A-allele.

*Supplementary Figure 6.* *Ugandan discovery sample.* Fitted probability values for lifetime posttraumatic stress disorder (PTSD) as a function of traumatic load are plotted separately for the three genotype groups of rs7700424 (chromosome 5). Results indicate an increased risk for PTSD development after traumatic experiences with increasing number of the minor C-allele.

*Supplementary Figure 7.* *Rwandan replication sample.* Fitted probability values for lifetime posttraumatic stress disorder (PTSD) as a function of traumatic load are plotted separately for the three genotype groups of rs3852144 (chromosome 5). As in the discovery sample, we see a decreased risk for PTSD development after traumatic experiences with increasing number of the minor G-allele.

*Supplementary Figure 8.* *Ugandan therapy sample*. Mean sum scores and standard errors of the Posttraumatic Stress Diagnostic Scale (PDS) before therapy (t_1_), 4 months after therapy (t_2_) and at 10-months follow up (t_3_), separately displayed for the three genotype groups of rs3852144 (chromosome 5). Post-hoc tests show a significant effect of genotype on the change in PDS symptom score from before treatment to the 4-months follow-up (comparison t_1_-t_2_), as well as between the 4- and the 10-months follow-up (comparison t_2_-t_3_). However, the symptom change from pre-treatment to the 10-months follow-up assessment (comparison t_1_-t_3_) was not influenced by genotype. Descriptively we see that this effect seems to depend mainly on the G/G-genotype group.

**References**

Bellenguez, C., Strange, A., Freeman, C., Wellcome Trust Case Control Consortium, Donnelly, P., & Spencer, C. C. (2012). A robust clustering algorithm for identifying problematic samples in genome-wide association studies. *Bioinformatics, 28*(1), 134-135. doi:10.1093/bioinformatics/btr599

Purcell, S., Neale, B., Todd-Brown, K., Thomas, L., Ferreira, M. A., Bender, D., . . . Sham, P. C. (2007). PLINK: A tool set for whole-genome association and population-based linkage analyses. *The American Journal of Human Genetics, 81*(3), 559-575. doi:10.1086/519795
